# Continuous Renal Replacement Therapy in Children

**DOI:** 10.1007/s13312-025-00026-4

**Published:** 2025-03-21

**Authors:** Maha Haddad, Lavjay Butani

**Affiliations:** Division of Pediatric Nephrology, Department of Pediatrics, University of California, Davis, 2516 Stockton Blvd, Sacramento, CA 95817 USA

**Keywords:** Acute kidney injury, Dialysis, Plasma exchange, Renal failure

## Abstract

Continuous renal replacement (CRRT) therapies are a valuable addition to the dialytic armamentarium, and that, through gentler fluid removal and solute clearance, are better tolerated in critically ill children who are in need of dialysis. CRRT is technically demanding and resource intensive, and its use can be associated with many complications. There are many different modalities of CRRT, each of which employ varying combinations of convective and diffuse solute removal and ultrafiltration. CRRT is performed using specialized devices with their own unique dialysis filters and options for different circuit anticoagulation modalities. Because of the complexities inherent in the choice of CRRT and the monitoring involved, CRRT must be performed an intensive care unit setting, using a multidisciplinary team approach.

## What is Continuous Renal Replacement Therapy (CRRT)

Simplistically put, continuous renal replacement therapy (CRRT) refers to a set of dialytic modalities, that provide continuous, slow and gentler dialysis (volume removal and solute clearance) compared to intermittent hemodialysis (IHD). These modalities, by virtue of the gentler fluid removal and osmotic shifts, are better tolerated from a hemodynamic perspective and reduce the risk of cerebral edema. CRRT is therefore preferred in critically ill patients with severe acute kidney injury (AKI), compared to IHD (1). While acute peritoneal dialysis is also feasible in critically ill children, its use has declined considerably due to technological advancements in CRRT, and because volume removal and solute clearance can be more precisely controlled with CRRT.

## Indications for CRRT

Continuous Renal Replacement Therapy is mainly indicated in the critically ill patient with severe AKI, with hemodynamic instability, to treat or prevent fluid overload (FO) and to provide solute clearance, as often seen in children with severe sepsis. CRRT is the preferred method of renal support in these patients as it allows for gentle fluid removal and solute clearance [1]. Critically ill patients often require parenteral nutrition, multiple and frequent blood products, and vasoactive and other medications, all of which contribute to a large volume load. Early start of CRRT before fluid overload (FO) becomes severe has been associated with better outcomes [2, 3]. Patients receiving extracorporeal life support (ECLS) therapy are another example where CRRT is often used to prevent severe FO [4]. While inline filters in the ECLS circuit offer an alternative option for slow continuous ultrafiltration, they provide negligible clearance and put patients at risk for electrolyte derangements and elevated blood urea nitrogen with prolonged use.

Lastly, other indications for CRRT include instances where rapid re-accumulation of toxic metabolic products is anticipated after IHD, such as with hyperammonemia [5] and tumor lysis syndrome, and in some drug intoxications [6].

## Practical Considerations

### Vascular Access

An appropriately sized vascular access catheter that allows for adequate blood flow and circuit survival is essential; ideally this consists of a double lumen catheter in the venous circulation. Both the size and location of the catheter affect circuit survival. The right internal jugular (IJ) is the ideal choice as it enters directly into the right atrium; the use of such catheters is associated with longer circuit survival in comparison to catheters in the femoral vein and the superior vena cava. The catheter tip should be superior to the cavo-atrial junction. Femoral veins are the second preferred choice as they are easily placed in children; the catheter tip should be in the inferior vena cava. Femoral lines and are usually longer than IJ catheters to ensure that the tip is in a satisfactory position; such longer catheters are associated with more resistance to flow and are more subject to the patient’s movements. Larger bore catheters are associated with better circuit survival in comparison to smaller diameter catheters as they allow lower resistance to flow [7, 8]. More recent data indicate that the left brachiocephalic vein is also a good option when accessed using a point care ultrasound particularly for very small babies due to its large caliber and the fact that it is non-collapsable. Subclavian catheters should be avoided to preserve the veins in case long-term dialysis is needed in the future. In addition, they are associated with a higher risk of thrombosis and insertion complications. Umbilical lines should be avoided due to poor flow and increased risk of thrombosis. Please refer to Table 1 for suggested pediatric lines based on patient weight.

**Table 1:** Recommended dialysis catheter size based on patient size

Patient size	Catheter size
Neonate	Dual-lumen 7 French
3–6 kg	Dual-lumen 7 French
6–15 kg	Dual-lumen 8 French
15–30 kg	Dual-lumen 9–10 French
> 30 kg	Dual or triple-lumen 11.5–12.5 French

### Circuit Prime

Blood priming may be required when the extracorporeal circuit volume is > 10% of the intravascular volume of the child, as is often the case in neonates and small infants. A packed red blood cell unit typically has a hematocrit of 70–80%. This has to be diluted to a goal that is appropriate for the patient. For most patients mixing the unit with 5% albumin or normal saline in 1:1 ratio will achieve the goal. Frequent blood priming can increase sensitization of the patient, making a future renal transplant more challenging; therefore consideration should be given to limiting exposure to blood from different donors, if possible.

### Anticoagulation

Running the circuit without anticoagulation is associated with shorter circuit life because of the risk of clotting within the circuit, even in the setting of sepsis. The main methods of anticoagulation are regional citrate anticoagulation, systemic heparinization and prostacyclin. If available, regional citrate anticoagulation is the preferred modality because of the lowest risk of complications and higher circuit life; it is, however, more labor intensive [9].

### Dialysis Prescription

**Fig. 1:**
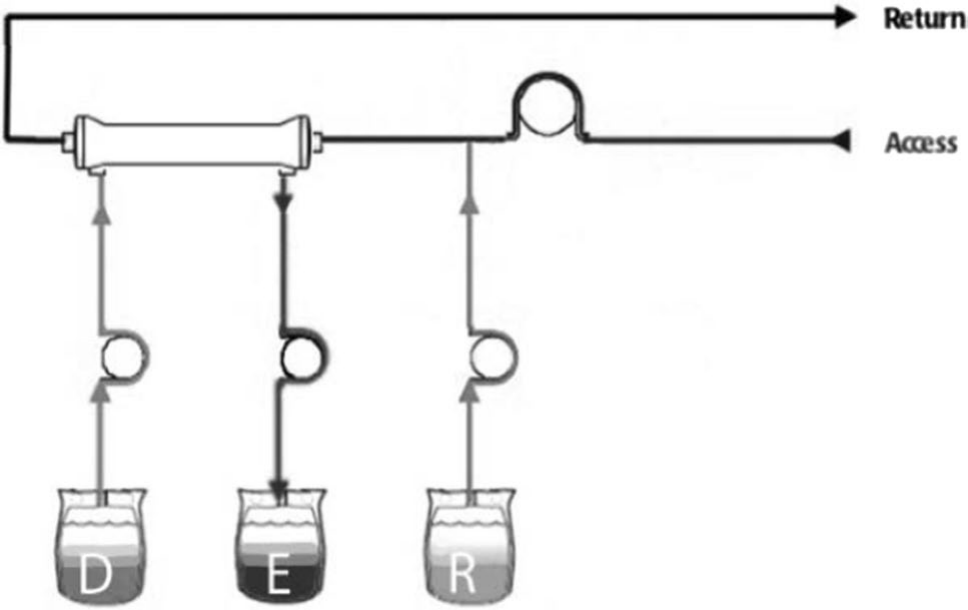
A schematic depicting different modalities of continuous renal replacement therapy. R, replacement fluid, E: effluent, D: dialysate. Slow Continuous Ultrafiltration: Movement of water molecules under the effect of hydrostatic pressure. No replacement fluid or dialysate are used. Negligible solute clearance. Continuous Veno-Venous Hemofiltration: Requires replacement fluid (R). Replacement fluid can be infused prefilter as shown in the diagram above, or postfilter. Solute clearance is through convective transport. Continuous Veno-Venous Hemodialysis: Only countercurrent dialysate (D) is used without replacement fluid. Solute clearance is by diffusion. Continuous Veno-Venous Hemodiafiltration: Both replacement fluid (R) and dialysate (D) are used simultaneously. Solute clearance occurs through a combination of diffusive and convective transport

Despite the wide use of CRRT in pediatric intensive care units, there is a wide variation in practice as it pertains to the precise modality of CRRT and the prescribed dialysis dose. The possible modalities of CRRT include slow continuous ultrafiltration (SCUF), continuous veno-venous hemofiltration (CVVH), continuous veno-venous hemodialysis (CVVHD) and continuous veno-venous hemodiafiltration (CVVHDF). SCUF involves pure ultrafiltration without the use of dialysate or replacement fluids. CVVHD uses diffusion for solute clearance. In this modality countercurrent dialysate is used, without the use of replacement fluids. CVVH mainly uses convection as a method of solute clearance. In CVVH, a replacement-fluid is used. Clearance is dependent of the rate of replacement fluids as well as membrane characteristics. Replacement fluid can be infused either pre or post filter. In CVVHDF, both dialysate and replacement fluids are used. Clearance is mostly dependent on dialysate flow rate as well as membrane characteristics. Please see Fig. 1 for a schematic of the different CRRT modalities.

Convective clearance provides superior middle molecule clearance in comparison to diffusive clearance and provides better clearance of proinflammatory molecules that accumulate in sepsis. However, studies have failed to prove a beneficial outcome of convective clearance over diffusive clearance [10]. Some studies, however, indicate that the use of high volume CVVH (> 70 ml/kg/h) may be associated with improved outcomes in children with sepsis [11].

Several different machines are available for use for CRRT in the United States, the most common of which is the Prismaflex (Baxter, USA) and its newer version, the PrisMax (Baxter, USA). All modalities of CRRT are possible with the use of these devices; additional features of the PrisMax (Baxter, USA) are a blood warmer (Thermax) and the ability to discard the ultrafiltrate directly without having to use an effluent bag. In recent years, a smaller sized CRRT filter (HF20), which is more suitable for use in neonates, became available for use with the PrisMax (Baxter, USA) device. The different filter options available with the PrisMax (Baxter, USA) are outlined in Table 2.

**Table 2:** PrisMax filter sets and characteristics

Filter set	Surface area (m^2^)	Priming volume (ml)	Blood flow range (ml/min)	Max QUF** (ml/hr)	Fluid gain/loss limit (ml/3 h)	Patient’s weight (kg)
HF20	0.2	58	20–100	QB 50 = 1020	60–150	0–12 kg
				QB 100 = 1440		
ST 60*	0.6	97 ml	50–180	QB100 = 2340	60–200	12–20 kg
		124 ml (with Thermax)		QB180 = 3360		
HF 1000	1.1	165	80–400	QB100 = 2640	100–400	> 20 kg
		192 (with Thermax)		QB400 = 7620		

*ST membranes are AN69 membranes that are surface coated with Polyethylene imine to prevent bradykinin release syndrome)

**QUF (Ultrafiltration Rate) = patient fluid removal + replacement flow rate + pre blood pump flow rate

Other available CRRT machines in the US that provide very accurate fluid balance include the Carpedium machine (Mozark Medical, USA) and Aquadex (Nuwellis, USA) increasing safety in the smallest of all children, especially neonates. Table 3 compares these two new devices.

**Table 3:** Characteristics of Aquadex and Carpediem CRRT machines

CRRT machine	Aquadex	Carpediem
Modality	SCUF or CVVH	SCUF, CVVH, CVVHD
ECV (ml)	33	33 and 41
QB (ml/min)	10–40	2–50
UF (ml/hr)	0–500	0–400 and 0–600
Accuracy	± 10 ml/hr	± 1 ml/hr
Max Duration	72 h	24 h
Bags	Any	2 L bags only

ECV, extracorporeal volume; QB, Blood flow rate; UF, ultrafiltration rate

The typical prescribed blood flow rate at initiation of CRRT in children is 3–5 ml/kg/min. For safe fluid removal, a net ultrafiltration rate of 0.5–2 ml/kg/h is typically targeted. Higher rates should be avoided to prevent hemodynamic instability. For solute clearance, most centers target an initial total solute clearance of 2000 ml/1.73m^2^ for children and 30 ml/kg for adults (12). However, the dose can be increased when greater clearance is required such as in neonates with hyperammonemia and in instances where citrate is expected to accumulate as in children with liver failure who are receiving regional citrate anticoagulation. The solute clearance dose is then divided equally into convective (hemofiltration) and diffusive (dialytic) clearance, if CVVHD is the prescribed modality. To achieve the targeted convective transport, pre and post dialyzer replacement fluids are often needed. The advantage of pre-filter replacement fluid is that it provides continuous flushing of the filter, reducing the risk of clotting. However, it decreases the dose of solute clearance through diffusion, as the blood entering the filter gets diluted by the pre-filter fluid. The post-filter replacement fluid on the other hand does not reduce the diffusive solute clearance but is associated with increased risk of filter clotting since it increases the amount of fluid that has to be removed by the dialyzer, by adding to the fluid being administered to the patient. As the ultrafiltration dose increases one has to pay attention to the filtration fraction (FF) (FF = ultrafiltration rate divided by [(1-hematocrit) X (blood flow rate + prefilter fluid rate)]. The FF should be kept below 25%; the higher the FF, the higher the risk of filter clotting as it leads to higher post filter hematocrit. To decrease the FF, one can increase the blood flow rate, decrease the ultrafiltration rate, use a higher prefilter replacement or increase diffusive transport by adding a dialytic component. While increasing diffusive clearance is not associated with a higher risk of circuit clotting, the prescriber should balance this with the possible beneficial outcomes of convective transport in children with sepsis, through the removal of proinflammatory cytokines.

In addition to hemodynamic instability, rapid electrolyte changes and osmotic shifts and circuit clotting with the associated blood loss, that have been alluded to previously, another potential complication of CRRT is hypothermia, particularly in infants. The temperature of the patient, therefore should be carefully monitored. Every effort should be taken to warm the patient through strategies such as the use of radiant warmers in babies, use of warming blankets, and by wrapping lines with commercially available warming sleeves that engulf the circuit lines. In CRRT machines that use volumetric balancing systems, wrapping the solutions is also recommended; for CRRT machines that rely on scale-based ultrafiltrate measurements this should avoided as it can interfere with the accuracy of fluid balance [12].

Last but not least, CRRT prescribers must take into account the clearance of medications and antibiotics as well as nutrients when prescribing CRRT and use a multidisciplinary approach to optimize drug dosing and nutrition in these critically ill children.

Weaning from CRRT is based on a variety of factors: hemodynamic stability, volume status, solute control, and the minimum daily fluid inputs. Typically, CRRT is maintained in patients requiring vasopressors because it is tolerated better for fluid removal than other modalities. In addition, assessment on the degree of kidney recovery are key to CRRT weaning. No clear guidelines or tools are available, although several variables have been evaluated in retrospective observational studies; these include hemodynamic stability and especially urine output; a urine output before CRRT cessation has been a consistent positive predictor of successful RRT discontinuation. In adult studies, a urine output of 400 ml/day or 8.5 ml/kg per 24 h without diuretics, pre-CRRT liberation and a 6 h urine output after CRRT were important predictors associated with successful CRRT weaning [13].

## Outcomes Data

As one can imagine, well- designed and controlled randomized trials of outcomes using different dialytic modalities in critically ill patients with severe AKI are challenging and often not feasible; the few studies that have been performed have shown controversial and conflicting outcome data, either when comparing CRRT to IHD and also when comparing different intensities of CRRT [14, 15]. The small number of children with severe AKI needing dialysis, at any given center, further hinders such studies in the pediatric population. Having stated that, a recent adult randomized trial found that the initiation of CRRT in adults with AKI, as compared to IHD, was associated with a significant reduction in the composite outcome of death or dependence on renal replacement therapy at 90 days [16]. Furthermore, data from the multicenter Prospective Pediatric CRRT (ppCRRT) registry have helped elucidate some predictive factors pertaining to circuit survival and patient survival (such as %FO mentioned previously) and guided treating nephrologists on best practices [17].

## Data Availability

Not applicable.

## Declarations

### Generative AI and AI-Assisted Technologies in the Writing Process

None used for the preparation of this manuscript.
